# Efficacy and safety of netarsudil/latanoprost fixed-dose combination vs. monotherapy in open-angle glaucoma or ocular hypertension: A systematic review and meta-analysis of randomized controlled trials

**DOI:** 10.3389/fmed.2022.923308

**Published:** 2022-08-01

**Authors:** Nachuan Luo, Xun Jiang, Meiqi Hao, Zige Fang, Yiping Wei, Wenxiong Zhang

**Affiliations:** ^1^Department of Ophthalmology, The Second Affiliated Hospital of Nanchang University, Nanchang, China; ^2^Jiangxi Medical College, Nanchang University, Nanchang, China; ^3^Department of Thoracic Surgery, The Second Affiliated Hospital of Nanchang University, Nanchang, China

**Keywords:** latanoprost, netarsudil, fixed-dose combination, glaucoma, meta-analysis, topical medication

## Abstract

**Objective:**

As monotherapy is insufficient for some patients, the existing fixed-dose combination (FDC) requires two or more daily administrations with declining adherence. The present study compared the efficacy and safety of netarsudil/latanoprost FDC with monotherapy of its individual components in patients with glaucoma.

**Methods:**

A systematic literature search was performed for studies comparing netarsudil/latanoprost fixed-dose combination (FDC) vs. monotherapy in patients with glaucoma. The primary endpoints included intraocular pressure (IOP), intraocular pressure reduction percentage (IOPR%) and adverse events (AEs).

**Results:**

Three randomized controlled trial studies (RCTs) involving 1,692 patients (FDC: 556, netarsudil: 577, latanoprost: 559) were included in this meta-analysis. FDC was more effective than netarsudil, with significantly lower diurnal IOP over three time points (8:00 a.m., 10:00 a.m., 4:00 p.m.), mean diurnal IOP (MD = −2.36 [−3.08, −1.63], *P* < 0.00001) and higher IOPR% (MD = 9.60 [7.86, 11.33], *P* < 0.00001). When comparing FDC with latanoprost, both mean diurnal IOP (MD = −1.64 [−2.05, −1.23], *P* < 0.00001) and diurnal IOP across 3 time points were significantly lower with FDC than with latanoprost, while FDC induced significantly higher IOPR% (MD = 6.09 [4.40, 7.77], *P* < 0.00001). Incidence of total AEs was similar between netarsudil and FDC, but higher with FDC than with latanoprost.

**Conclusion:**

Netarsudil/latanoprost FDC appears to be superior to netarsudil or latanoprost alone, with better ocular hypotensive effects. However, there are concerns that netarsudil/latanoprost FDC was associated with a significantly higher incidence of AEs specifically compared with latanoprost.

**Systematic review registration:**

https://www.crd.york.ac.uk/PROSPERO/display_record.php?RecordID=311956.

## Introduction

Glaucoma has long been regarded as a major eye disease that can cause blindness (1). As the first line of treatment for glaucoma, topical medications lower the intraocular pressure (IOP) to delay damage to the optic nerve caused by elevated IOP (2). These include common agents such as β-adrenergic receptor antagonists, prostaglandin (PG) analogs, carbonic anhydrase inhibitors, adrenergic receptor agonists, rho-kinase inhibitors, and cholinergic agonists (3). Because of once-daily dosing convenience and effective IOP reduction, prostaglandin analogs are commonly prescribed as a preferred agent for patients with glaucoma (4). The main mechanism of its hypotensive effect is the increase in uveoscleral outflow and/or decrease in the production of aqueous humor (5). Clinical trials have been conducted with Rho kinase inhibitors as potential ocular hypotensive drugs for patients with open-angle glaucoma (OAG) or ocular hypertension (OHT), one of which, netarsudil (AR-13324), is the first product of a new generation of IOP-lowering agents to inhibit the norepinephrine transporter and rho kinase in the same compound (6). Rho kinase inhibitors reduce IOP by enhancing outflow facilitation (7). Additionally, the inhibition of the norepinephrine transporter may also play an important role by decreasing the production of aqueous humor (8).

Nevertheless, monotherapy is insufficient for many patients to achieve target IOP, which necessitates the use of multiple drugs (9). As a result of polypharmacy's increased complexity, medication adherence often decreases, which may negatively impact clinical outcomes. Moreover, monotherapy requires a higher dose to reach a target IOP, which may exacerbate adverse events (AEs), such as latanoprost-mediated increases in iris and skin pigments (10). Fixed-dose combination (FDC) formulations of IOP-lowering medications may meet the need for greater efficacy than monotherapy. However, the currently available FDCs for glaucoma do not contain rho-kinase inhibitors. A novel FDC product containing latanoprost and netarsudil with the benefit of once-daily dosing, also known as Rocklatan^TM^, recently approved by the FDA (US Food and Drug Administration), has already been evaluated to solve the current problem in the former study, but controversies remain (11).

Thus, we conducted a meta-analysis of related randomized controlled trial studies (RCTs) to compare the IOP-lowering efficacy and safety of netarsudil/latanoprost FDC with its individual components for treating patients with OAG or OHT.

## Materials and methods

PRISMA (Preferred Reporting Items for Systematic Review and Meta-Analysis) guidelines were followed in the conduct of this meta-analysis. (PROSPERO registration number: CRD42022311956).

### Search strategy

Relevant articles were retrieved using the following electronic databases: (1) EMBASE; (2) Ovid MEDLINE; (3) PubMed; (4) Web of Science; (5) ScienceDirect; (6) The Cochrane Library; and (7) Google Scholar. The following terms were used: “glaucoma,” “Netarsudil,” and “Latanoprost.” The last search was on January 25th, 2022. The detailed search results are shown in Supplementary Table 1. Additional qualified studies were acquired by searching the references of the retrieved articles.

### Inclusion and exclusion criteria

Study eligibility criteria for inclusion in this meta-analysis are listed as follows. (1) Population: patients with OAG or OHT; (2) Intervention and comparison: netarsudil/latanoprost FDC vs. monotherapy (latanoprost or netarsudil) once daily; (3) Outcome parameters: diurnal IOP over the three time points (8:00 a.m., 10:00 a.m., and 4:00 p.m.), mean diurnal IOP, IOP reduction percentage (IOPR%), AEs; (4) Study design: only RCTs published in English were included. If two or more articles were from the same population, we selected the paper published most recently. In addition, abstracts only, animal research, articles with duplicated data, meta-analyses, and review articles with no original data were excluded.

### Outcome measures

The following outcomes were recorded as the primary outcome measures for the efficacy of the medications: diurnal IOP over the three time points (8:00 a.m., 10:00 a.m., and 4:00 p.m.), mean diurnal IOP and IOPR% from baseline to the endpoint. We utilized existing IOPR% data directly if they were available in the original study. If not, we applied the following principles for calculation: IOPR = IOP_baseline_ − IOP_end-*point*_, SD_IOPR_ = (${\text{SD}}_{\text{baseline}}^{2}$ + ${\text{SD}}_{\text{end}\text{-}point}^{2}$ − SD_baseline_ × SD_end-*point*_)^1/2^, while the IOPR% and SD of the IOPR% (SD_IOPR%_) were estimated by IOPR% = IOPR/IOP_baseline_, SD_IOPR%_ = SD_IOPR_/IOP_baseline_ (12). The eye with the highest baseline pressure or the right eye in the event of a tie was determined as the study eye by the authors of primary studies, as described previously (8, 11). For the safety assessment, the proportions of patients with adverse events were considered, which can be classified into four main groups by system organ class (general disorders and administration site conditions, eye disorders, infections and infestations, investigations).

### Data extraction

All data were collected by two investigators independently. The information included the article characteristics (country, first author, publication year), participant characteristics (number of patients, age, sex, study eye diagnosis, race and iris color), duration of follow-up, IOP measurements and number of AEs. Discussions were held to resolve any disagreements.

### Quality assessment

The RCTs were evaluated for methodological quality with the risk-of-bias tool in the Cochrane Handbook for Systematic Reviews of Interventions (version 5.1.0) (13). All included studies were evaluated for detection bias, performance bias, reporting bias, selection bias, attrition bias, and other biases. The 5-point Jadad scale was also used for quality assessment, which includes 3 main aspects: accountability of all patients, masking and randomization. Study scores ≥3 points were defined as high-quality (14).

We examined the quality of evidence for the outcomes based on the Grading of Recommendations Assessment, Development and Evaluation (GRADE) guidelines, which consider risk of bias, publication bias, indirectness, inconsistency and imprecision. The evidence was divided into high, medium, low, or very low levels (15).

### Statistical analysis

We analyzed the data with the Stata software package (version 15.1; Stata Corp., College Station, TX) and Cochrane Review Manager (RevMan, software version 5.3, Copenhagen, Denmark: The Nordic Cochrane Center, The Cochrane Collaboration, 2014). Comparisons between FDC and monotherapy were grouped by the component of monotherapy (FDC vs. netarsudil and FDC vs. latanoprost). Subgroup analysis of IOPR% was conducted to determine whether the results would change according to follow-up duration, loss to follow-up rate, nation, number of patients, publication year, and trial center. The weighted mean difference (WMD) was measured for continuous variables (IOP across the 3 diurnal time points, mean diurnal IOP and IOPR%), while the risk ratio (RR) was estimated for dichotomous variables such as AEs. The outcomes were all presented with a 95% confidence interval (CI). *P* < 0.05 indicates statistical significance. Heterogeneity in this study was examined with the *I*^2^ statistic and χ^2^ test. If the heterogeneity was acceptable (*P* > 0.1, *I*^2^ < 50%), the fixed-effects model was adopted. If not, a random-effects model was employed. We inspected publication bias with Begg's and Egger's tests (16, 17).

## Results

### Study characteristics and quality assessment

**Figure 1:**
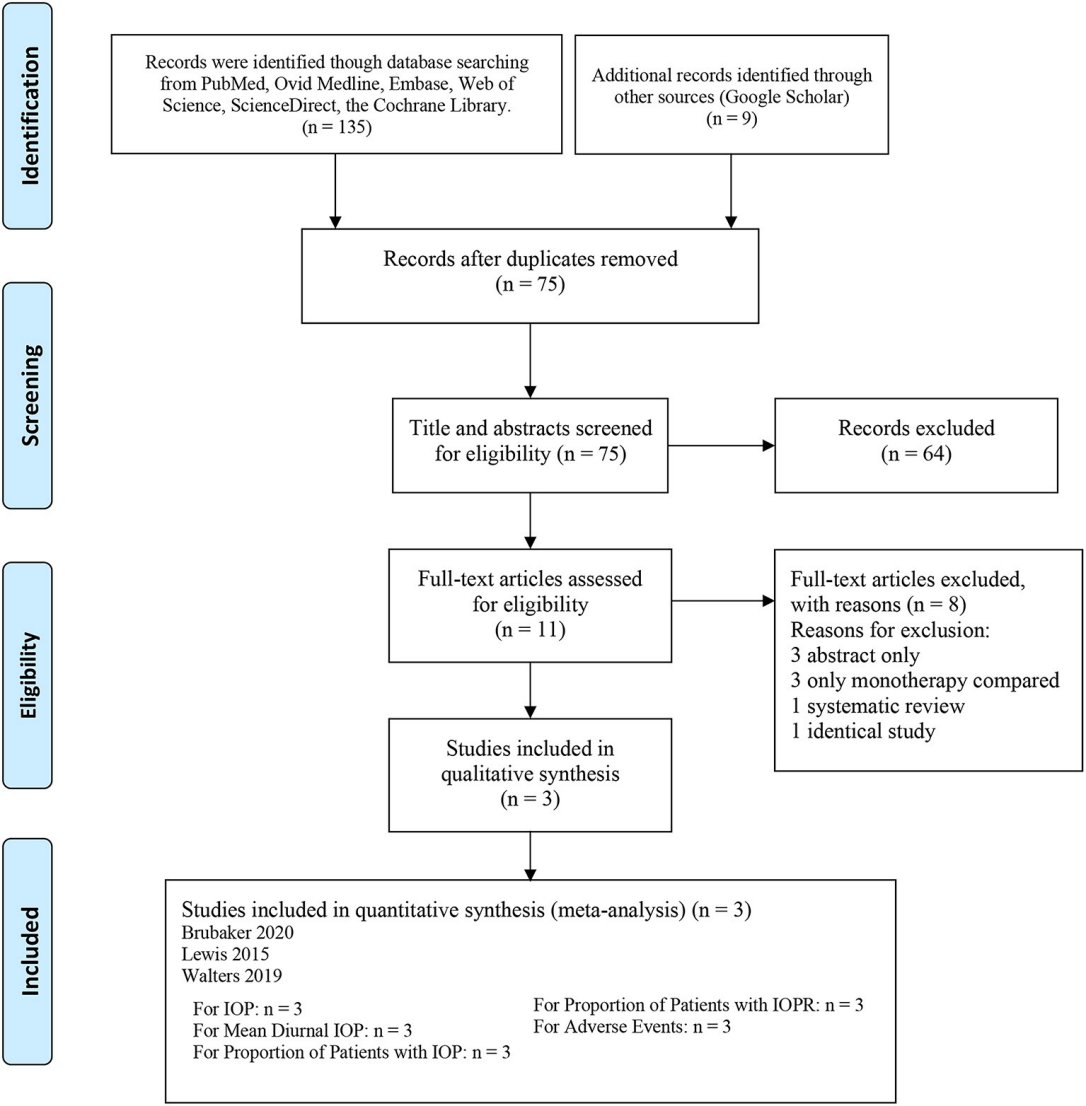
Flow chart of study selection.

In all, 144 articles were initially identified. After applying the exclusion criteria, 3 articles were included in the present meta-analysis, as shown in Figure 1 (18–20). In total, 1,692 patients participated in this systematic review, which included 556 patients in the netarsudil/latanoprost FDC group, 577 patients in the netarsudil group, and 559 patients in the latanoprost group. Overall, the included RCTs demonstrated a low risk of bias for the majority of domains evaluated (Supplementary Figure 1). According to the 5-point Jadad scale, all 3 RCTs were of high quality (Supplementary Table 2). A summary of the characteristics and main assessments of the included studies is shown in Table 1. Meanwhile, all of the outcome indicators for the study design and therapeutic strategy for the efficacy and safety outcomes were assessed as high, moderate or low levels of GRADE quality (Supplementary Table 3).

**Table 1:** Summary of the baseline characteristics of the included studies.

**References**	**Nation**	**Follow-up** ** (month)**	**Groups**	**No. of patients**	**Sex** ** (M/F)**	**Age (Mean, year)**	**Study eye diagnosis**		**Race**						**Iris color**			
							**Ocular** ** hypertension**	**Open angle glaucoma**	**Asian**	**Black or African American**	**Native American**	**White**	**Multiple**	**Other**	**Blue/** **Gray/** **Green**	**Brown/** **Black**	**Hazel**	**Other**
Brubaker et al. (18)	USA	12	FDC^a^	238	104/134	64.4	65	173	7	69	0	162	0	0	68	141	29	0
			Netarsudil	244	108/136	64.6	57	187	6	70	0	167	1	0	73	137	34	0
			Latanoprost	236	100/136	65.4	55	181	10	67	0	157	2	0	62	154	20	0
Lewis et al. (19)	USA	1	FDC	73	34/39	64.2			1	10	0	62	0	0	24	39	10	0
			Netarsudil	78	35/43	64.8	NA	NA	2	17	1	58	0	0	20	50	8	0
			Latanoprost	73	27/46	65.1			1	12	0	60	0	0	14	48	11	0
Walters et al. (20)	USA and Canada	3	FDC	245	152/93	64.2	72	172	7	74				3	49	172	24	
			Netarsudil	255	153/102	64.5	68	187	11	76	NA	NA	NA	3	48	185	22	NA
			Latanoprost	250	144/106	64.3	79	171	6	79				2	52	174	24	

*FDC, fixed-dose combination; M/F, male/female; NA, not applicable*.

a *FDC: fixed-dose combination of Netarsudil/Latanoprost was applied*.

### Netarsudil/latanoprost FDC vs. netarsudil

**Figure 2:**
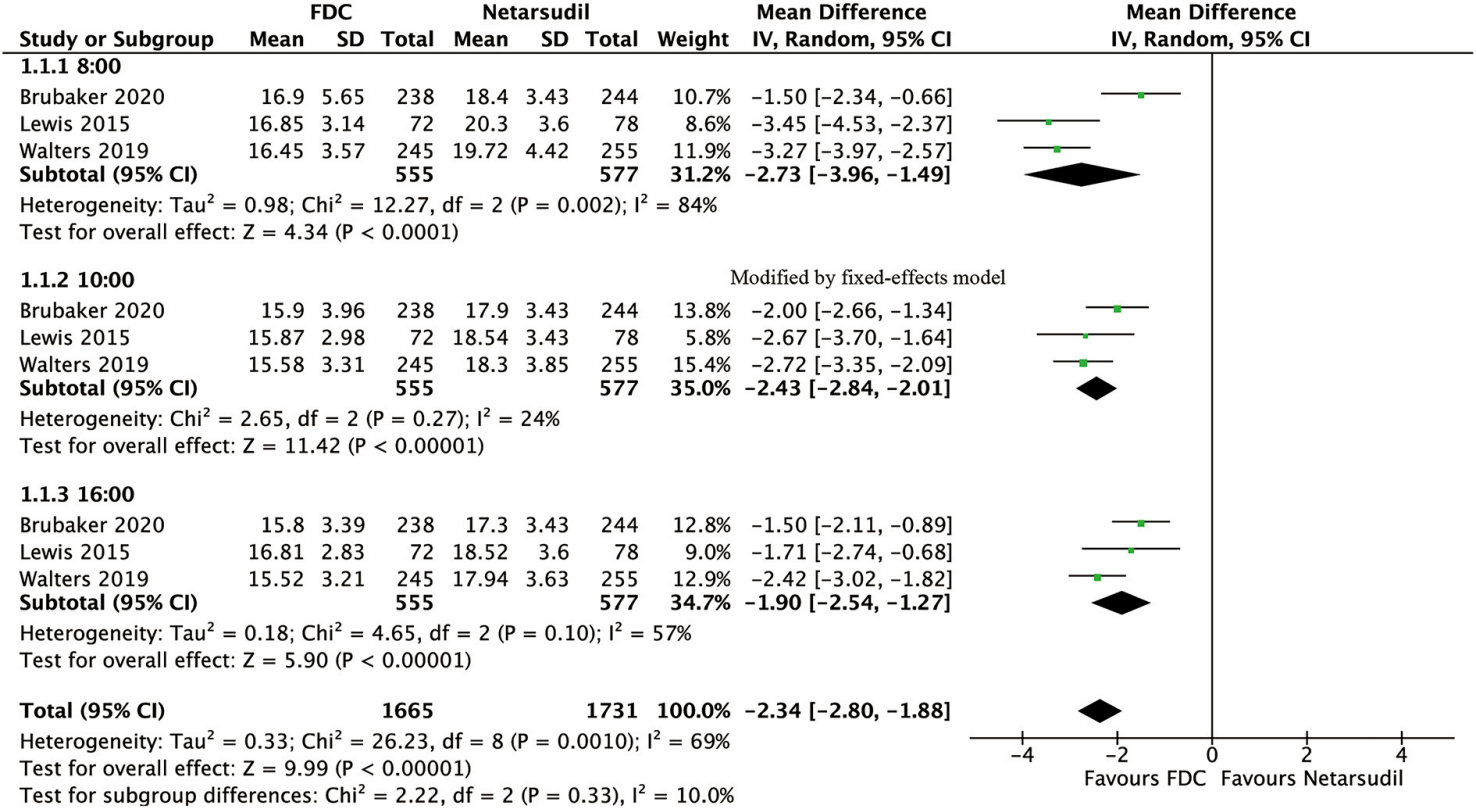
Forest plot of WMD of IOP at 3 time points (8:00 a.m., 10:00 a.m., 4:00 p.m.) associated with FDC vs. netarsudil.

**Figure 3:**
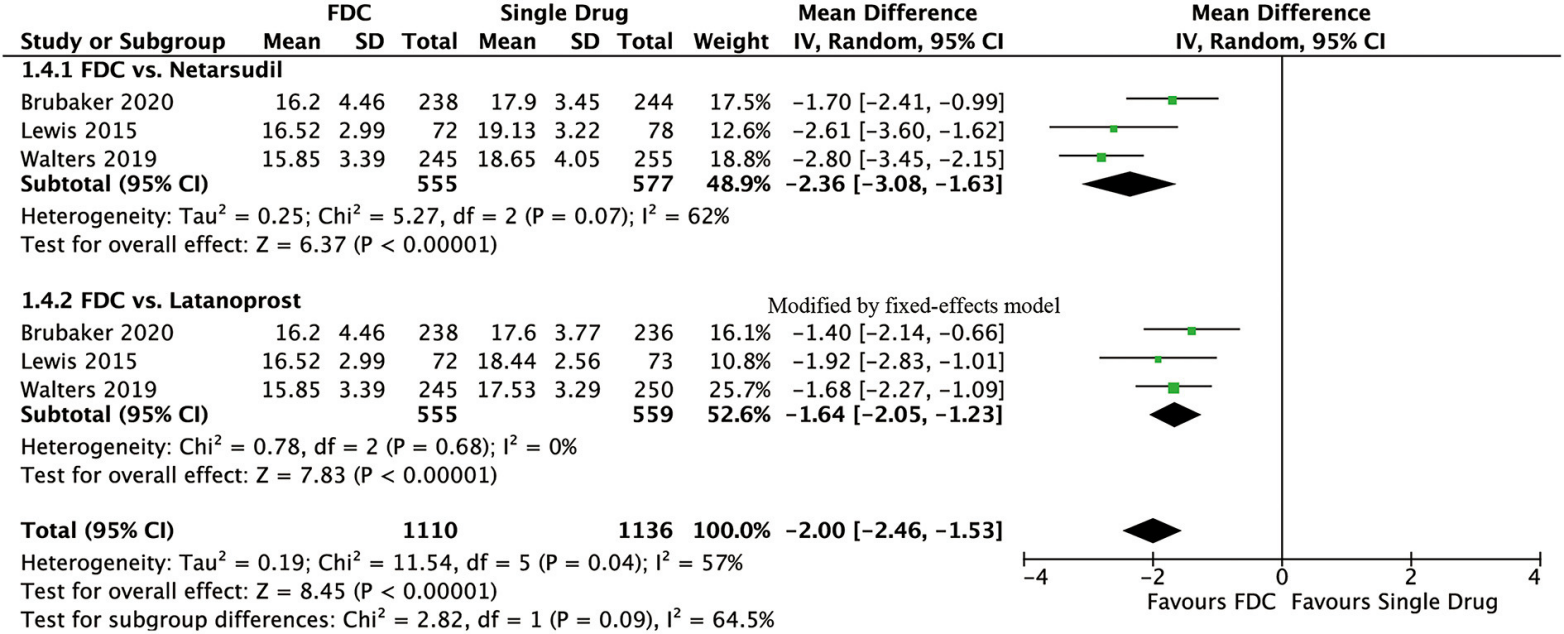
Forest plot of WMD of mean diurnal IOP associated with FDC vs. monotherapy.

**Figure 4:**
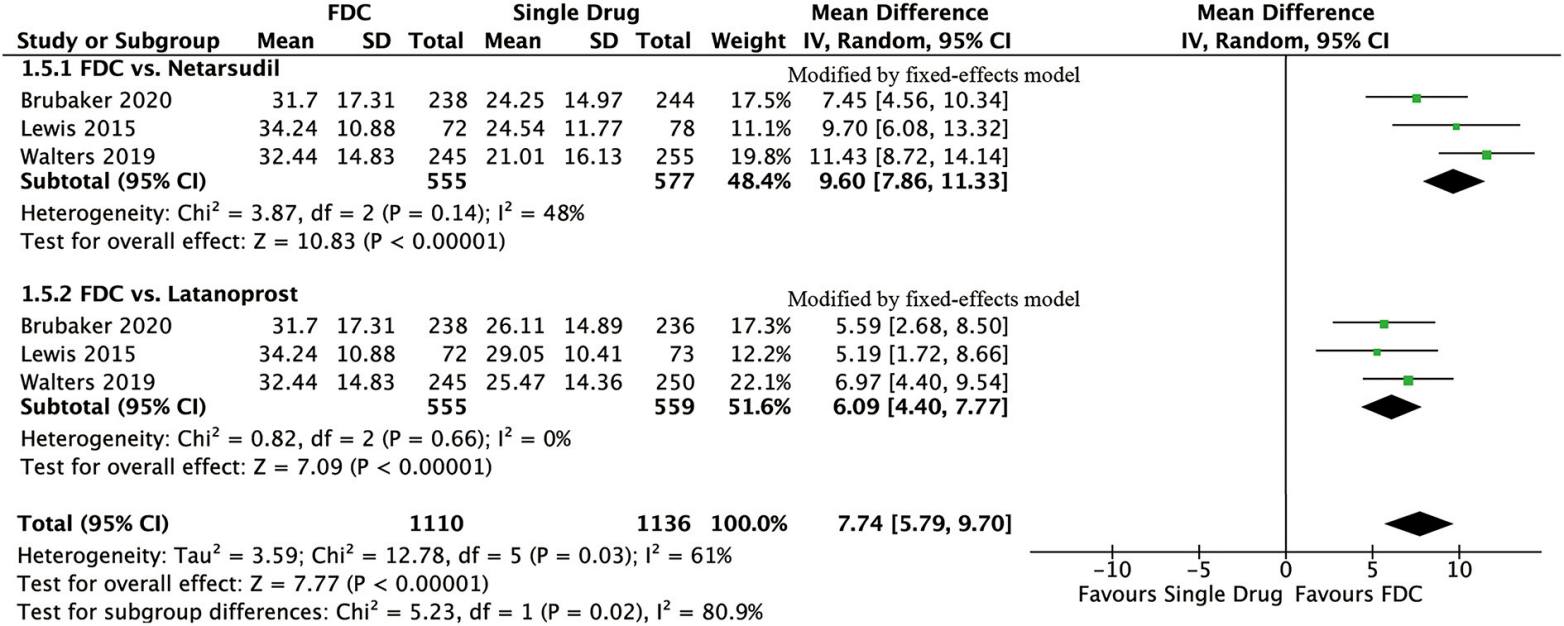
Forest plot of WMD of IOPR% associated with FDC vs. monotherapy.

For efficacy, IOP was significantly lower in the FDC group than in the netarsudil group across all 3 diurnal time points (8:00 a.m.: MD = −2.73 [−3.96, −1.49], *P* < 0.0001, *I*^2^ = 84%; 10:00 a.m.: MD = −2.43 [−2.84, −2.01], *P* < 0.00001, *I*^2^ = 24%; 4:00 p.m.: MD = −1.90 [−2.54, −1.27], *P* < 0.00001, *I*^2^ = 57%, Figure 2), as well as the mean diurnal IOP (MD = −2.36 [−3.08, −1.63], *P* < 0.00001, *I*^2^ = 62%, Figure 3). Moreover, IOPR% was in favor of FDC rather than netarsudil (MD = 9.60 [7.86, 11.33], *P* < 0.00001, *I*^2^ = 48%; Figure 4). In the subgroup analysis of IOPR%, no inconsistency was identified (Table 2).

**Table 2:** Subgroup analysis for percentage intraocular pressure reduction (IOPR%).

**Group**	**FDC vs. netarsudil**				**FDC vs. latanoprost**			
	**No. of studies**	**WMD (95% CI)**	***P***	*I*^2^ **(%)**	**No. of studies**	**WMD (95% CI)**	***P***	***I**^2^* **(%)**
Total	3	9.60 (7.86–11.33)	<0.00001	48	3	6.09 (4.40–7.77)	<0.00001	0
**Follow-up (month)**								
<1	0	-	-	-	0	-	-	-
1~6	2	10.81 (8.64–12.98)	<0.00001	0	2	6.34 (4.27–8.40)	<0.00001	0
6~12	1	7.45 (4.56–10.34)	<0.00001	-	1	5.59 (2.68–8.50)	0.0002	-
**Loss to follow-up rate**								
<10%	2	10.81 (8.64–12.98)	<0.00001	0	2	6.34 (4.27–8.40)	<0.00001	0
≥10%	1	7.45 (4.56–10.34)	<0.00001	-	1	5.59 (2.68–8.50)	0.0002	-
**Nation**								
USA	3	9.60 (7.86–11.33)	<0.00001	48	3	6.09 (4.40–7.77)	<0.00001	0
Canada	1	11.43 (8.72–14.14)	<0.00001	-	1	6.97 (4.40–9.54)	<0.00001	-
**Patients**								
<300	1	9.70 (6.08–13.32)	<0.00001	-	1	5.19 (1.72–8.66)	0.003	-
300~500	0	-	-	-	0	-	-	-
≥500	2	9.47 (5.57–13.37)	<0.00001	74	2	6.36 (4.44–8.29)	<0.00001	0
**Publication year**								
2019~2022 (last 3 years)	2	9.47 (5.57–13.37)	<0.00001	74	2	6.36 (4.44–8.29)	<0.00001	0
2017~2022 (last 5 years)	2	9.47 (5.57–13.37)	<0.00001	74	2	6.36 (4.44–8.29)	<0.00001	0
2012~2022 (last 10 years)	3	9.60 (7.86–11.33)	<0.00001	48	3	6.09 (4.40–7.77)	<0.00001	0
**Trial Center**								
Single	1	9.70 (6.08–13.32)	<0.00001	-	1	5.19 (1.72–8.66)	0.003	-
Multiple	2	9.47 (5.57–13.37)	<0.00001	74	2	6.36 (4.44–8.29)	<0.00001	0

*FDC, fixed-dose combination; IOPR%, percentage intraocular pressure reduction; No., number; RR: relative risk; VS., versus; WMD, weighted mean difference; “-”, not available*.

For safety, two studies compared total adverse events (heterogeneity: *P* = 0.44, *I*^2^ = 0%). We did not observe any significant difference between the two groups (RR = 1.04, 95% CI: 0.96–1.13, *P* = 0.33; Supplementary Figure 2). Although the comparison of 3 system organ classes for AEs showed no significant difference between the FDC group and the netarsudil group (general disorders and administration site conditions: RR = 1.21 [0.99, 1.48], *P* = 0.06, *I*^2^ = 24%, Supplementary Figure 3; investigations: RR = 0.74 [0.36, 1.53], *P* = 0.42, *I*^2^ = 0%, Supplementary Figure 4; infections and infestations: RR = 0.53 [0.10, 2.83], *P* = 0.46, Supplementary Figure 5), the netarsudil group had a significantly lower incidence rate of eye disorders than the FDC group (RR = 1.13 [1.05, 1.21], *P* = 0.001, *I*^2^ = 22%; Supplementary Figure 6). Among eye disorders, conjunctival hyperemia, cornea verticillate and conjunctival hemorrhage were the most frequent ocular AEs. General disorders and administration site conditions mainly manifested as instillation site erythema and instillation site pain/discomfort (Table 3).

**Table 3:** Comparison of adverse events grouped by SOC between Netarsudil/Latanoprost FDC and Netarsudil.

**SOC**	**Studies involved**	**FDC**		**Single drug**		**Total incidence %**	**Risk ratio**	**95% CI**	***I**^2^* **(%)**	***P***
		**Event/total**	**%**	**Event/total**	**%**					
Eye disorders	3	423/555	76.22	389/576	67.53	71.79	1.13	1.05–1.21	22	0.001
Conjunctival hyperemia	3	312/555	56.22	265/576	46.01	51.02	1.22	1.09–1.37	0	0.0007
Cornea verticillata	2	74/482	15.35	58/498	11.65	13.47	1.32	0.96–1.81	0	0.09
Conjunctival hemorrhage	3	57/555	10.27	77/576	13.37	11.85	0.76	0.56–1.05	0	0.10
Eye pruritus	2	31/311	9.97	24/321	7.48	8.70	1.33	0.80–2.20	0	0.28
Increased lacrimation	2	21/311	6.75	25/321	7.79	7.28	0.87	0.49–1.51	0	0.61
Punctate Keratitis	1	12/238	5.04	18/243	7.41	6.24	0.68	0.34–1.38	-	0.29
Visual acuity reduced	1	13/238	5.46	13/243	5.35	5.40	1.02	0.48–2.16	-	0.96
Vision blurred	1	11/238	4.62	15/243	6.17	5.40	0.75	0.35–1.60	-	0.45
Corneal disorder	1	14/244	5.74	12/255	4.71	5.21	1.22	0.58–2.58	-	0.60
Blepharitis	1	15/238	6.30	8/243	3.29	4.78	1.91	0.83–4.43	-	0.13
General disorders and administration site conditions	3	154/555	27.75	132/576	22.92	25.29	1.21	0.99–1.48	24	0.06
Instillation site erythema	1	14/73	19.18	17/78	21.79	20.53	0.88	0.47–1.65	-	0.69
Instillation site pain/discomfort	3	120/555	21.62	103/576	17.88	19.72	1.27	0.82–1.96	60	0.29
Investigations	2	12/317	3.79	17/333	5.11	4.46	0.74	0.36–1.53	0	0.42
Vital dye staining cornea present	1	10/244	4.10	14/255	5.49	4.81	0.75	0.34–1.65	-	0.47
Infections and infestations	1	2/73	2.74	4/78	5.13	3.97	0.53	0.10–2.83	-	0.46

*CI, confidence interval; FDC, fixed-dose combination; SOC, system organ class*.

### Netarsudil/latanoprost FDC vs. latanoprost

**Figure 5:**
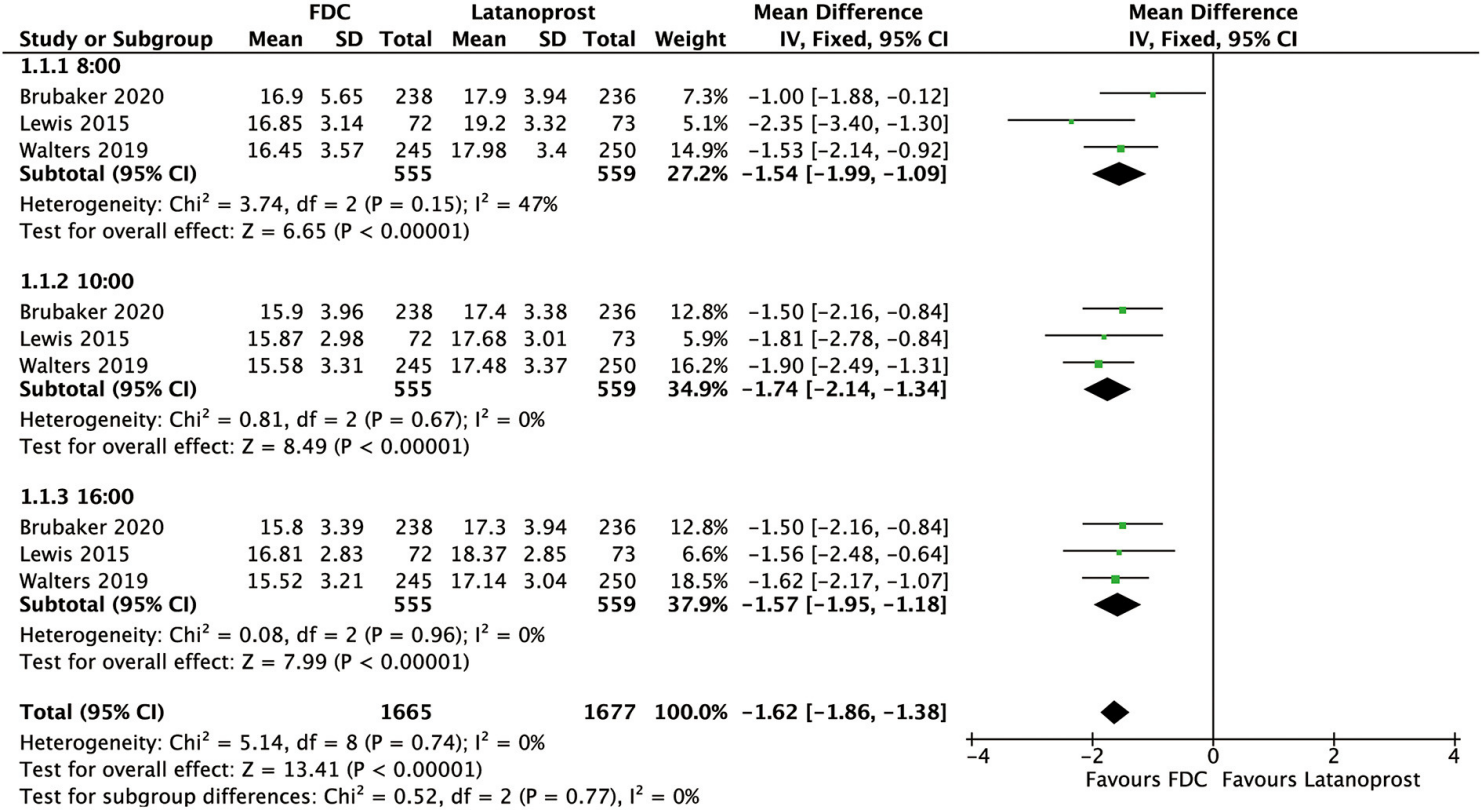
Forest plot of WMD of IOP at 3 time points (8:00 a.m., 10:00 a.m., 4:00 p.m.) associated with FDC vs. latanoprost.

For efficacy, IOP across three time points (8:00 a.m.: MD = −1.54 [−1.99, −1.09], *P* < 0.00001, *I*^2^ = 47%; 10:00 a.m.: MD = −1.74 [−2.14, −1.34], *P* < 0.00001, *I*^2^ = 0%; 4:00 p.m.: MD = −1.57 [−1.95, −1.18], *P* < 0.00001, *I*^2^ = 0%; Figure 5), mean diurnal IOP (MD = −1.64 [−2.05, −1.23], *P* < 0.00001, *I*^2^ = 0%) and IOPR% (MD = 6.09 [4.40, 7.77], *P* < 0.00001, *I*^2^ = 0%) were in favor of FDC rather than latanoprost (Figures 3, 4). The results of the subgroup analysis remained constant (Table 2).

For safety, FDC had a significantly higher incidence of total AEs than latanoprost (RR = 1.81 [1.22, 2.69], *P* < 0.003, *I*^2^ = 78%; Supplementary Figure 2). Compared with the FDC group, lower incidences of eye disorders (RR = 2.63 [1.84, 3.74], *P* < 0.00001, *I*^2^ = 81%; Supplementary Figure 6) as well as general disorders and administration site conditions (RR = 3.16 [1.84, 5.42], *P* < 0.0001, *I*^2^ = 61%; Supplementary Figure 3) were demonstrated in the latanoprost group, as the incidences of investigations (RR = 1.36 [0.58, 3.19], *P* = 0.47, *I*^2^ = 0%; Supplementary Figure 4) together with infections and infestations (RR = 0.50 [0.09, 2.65], *P* = 0.41; Supplementary Figure 5) were similar between groups. The eye disorders with the highest frequency were conjunctival hyperemia, cornea verticillate and eye pruritus. Moreover, except for reduced visual acuity and punctate keratitis, the other eye disorders showed a significantly higher incidence in the FDC group than in the latanoprost group. For general disorders and administration site conditions, FDC also induced significantly higher rates of instillation site pain/discomfort and instillation site erythema (Table 4).

**Table 4:** Comparison of adverse events grouped by SOC between netarsudil/latanoprost FDC and latanoprost.

**SOC**	**Studies involved**	**FDC**		**Single drug**		**Total incidence %**	**Risk ratio**	**95% CI**	*I*^2^ **(%)**	***P***
		**Event/total**	**%**	**Event/total**	**%**					
Eye disorders	3	423/555	76.22	159/561	28.34	52.15	2.63	1.84–3.74	81	<0.00001
Conjunctival hyperemia	3	312/555	56.22	118/561	21.03	38.53	2.67	2.24–3.19	0	<0.00001
Cornea verticillata	2	74/482	15.35	0/488	0.00	7.63	2.59	1.94–3.46	0	<0.0001
Eye pruritus	2	31/311	9.97	5/310	1.61	5.80	4.76	1.09–20.81	53	0.04
Conjunctival hemorrhage	3	57/555	10.27	5/561	0.89	5.56	10.54	4.43–25.06	0	<0.00001
Punctate keratitis	1	12/238	5.04	10/237	4.22	4.63	1.19	0.53–2.71	-	0.67
Blepharitis	1	15/238	6.30	5/237	2.11	4.21	2.99	1.10–8.09	-	0.03
Visual acuity reduced	1	13/238	5.46	6/237	2.53	4.00	2.16	0.83–5.58	-	0.11
Increased lacrimation	2	21/311	6.75	2/310	0.65	3.70	1.59	0.99–2.55	0	0.001
Vision blurred	1	11/238	4.62	3/237	1.27	2.95	3.65	1.03–12.92	-	0.04
Corneal disorder	1	14/244	5.74	0/251	0.00	2.83	29.83	1.79–497.29	-	0.02
General disorders and administration site conditions	3	154/555	27.75	54/561	9.63	18.64	3.16	1.84–5.42	61	<0.0001
Instillation site pain/discomfort	3	120/555	21.62	37/561	6.60	14.07	3.28	2.31–4.65	0	<0.00001
Instillation site erythema	1	14/73	19.18	1/73	1.37	10.27	14.00	1.89–103.72	-	0.010
Investigations	2	12/317	3.79	9/324	2.78	3.28	1.36	0.58–3.19	0	0.47
Vital dye staining cornea present	1	10/244	4.10	7/251	2.79	3.43	1.47	0.57–3.80	-	0.43
Infections and infestations	1	2/73	2.74	4/73	5.48	4.11	0.50	0.09–2.65	-	0.41

*CI, confidence interval; FDC, fixed-dose combination; SOC, system organ class*.

### Sensitivity analysis

In the analysis of IOP across 3 diurnal time points, mean diurnal IOP and IOPR% for FDC vs. monotherapy, significant heterogeneity was identified. To assess the stability and sensitivity, each study was assessed for its impact on pooled results. A sensitivity analysis of mean diurnal IOP and IOPR% demonstrated that the ultimate outcomes were reliable and robust (Supplementary Figure 7).

### Publication bias

The funnel plot analysis indicated no definitive evidence for publication bias in the comparison of mean diurnal IOP or IOPR% between the FDC group and monotherapy group. Furthermore, Begg's and Egger's tests did not detect any publication bias (Supplementary Figure 8).

## Discussion

For many glaucoma patients, current monotherapy with topical IOP-lowering agents is not sufficiently effective to achieve target IOP (21). However, most commercially available FDCs for glaucoma, including Cosopt® (timolol-dorzolamide), Combigan® (timolol-brimonidine) and Simbrinza® (brinzolamide-brimonidine), require at least twice-daily dosing, which greatly reduces compliance and adherence to the treatment (22–24). Although a novel FDC of netarsudil and latanoprost meets the convenience of once-daily administration, both its efficacy and safety remain unclear. This was the first meta-analysis to evaluate the ocular hypotensive effect and safety of netarsudil/latanoprost FDC compared with monotherapy. In the present study, we reviewed 3 RCTs in total. Netarsudil/latanoprost FDC appears to be better than netarsudil, with a better IOP reduction efficacy and a similar incidence of AEs. Although netarsudil/latanoprost FDC can achieve a better IOP-lowering effect than latanoprost, it is related to a significantly higher incidence of AEs with acceptable safety.

As seen from the efficacy analysis, the IOP across different time points and the mean diurnal IOP in the FDC group were significantly lower than those in each monotherapy group. Additionally, the IOPR% was in favor of FDC rather than monotherapy. Similar results were observed in a previous study (11). Moreover, we did not detect any inconsistency in the subgroup analysis. Walters' study also reported that netarsudil/latanoprost FDC could lower IOP by 3 mmHg more than monotherapy of its individual components (20). All of these superior IOP reduction effects could contribute to the increase in trabecular outflow due to netarsudil and uveoscleral outflow due to latanoprost (25). As a PG F_2α_ analog, latanoprost also decreases IOP through decreasing trabecular outflow resistance which is subsidiary. Furthermore, apart from increasing the trabecular outflow, decrease in aqueous humor production and reduction in episcleral venous pressure also play an important role in lowering IOP induced by netarsudil (26). Since it is universally acknowledged that increased resistance to AH outflow is the major factor that causes elevated IOP, increasing the trabecular outflow is regarded as the most physio-logical way of IOP reduction. Consequently, the fact that netarsudil lowers IOP through several different mechanisms of action may provide additional IOP lowering when combined with latanoprost (11, 26). Thus, netarsudil/latanoprost FDC could not only achieve the targeted IOP level but also have the convenience of once-daily dosing. These consistent results provide firm evidence for the wide range of indications for netarsudil/latanoprost FDC, considering its adequate ocular hypotensive effect.

The evaluation of safety indicated a similar incidence of total AEs in the FDC group compared with netarsudil, but FDC was relevant to a significantly higher incidence of AEs in the FDC vs. latanoprost groups. Similar conclusions have been drawn in several prior studies (11, 18–20). Among all eye disorders, conjunctival hyperemia, cornea verticillata, and conjunctival hemorrhage were the most frequent. We found that FDC resulted in a significantly higher incidence of conjunctival hyperemia than both latanoprost and netarsudil, which was likely related to vasodilation instead of irritation (27). Previous studies have demonstrated that latanoprost may induce mild conjunctival hyperemia (28). Nevertheless, FDC resulted in a higher incidence of hyperemia than netarsudil or latanoprost used alone, which may suggest a synergistic effect. A previous animal experiment in dogs showed that the unilateral administration of netarsudil resulted in bilateral conjunctival hyperemia (29). Thus, we assumed that the mechanism of enhanced hyperemia may be interpreted as the increased systemic absorption induced by netarsudil in FDCs. The significantly higher incidence of cornea verticillate was only observed with FDC compared with latanoprost instead of netarsudil, which suggested that it may be related to the netarsudil component. According to Lin's study, netarsudil, as a cationic amphiphilic drug, could cause phospholipidosis in Chinese hamster ovary cells, which indicated that the process of the accumulation of phospholipids within lysosomes of corneal epithelial cells may account for netarsudil-associated cornea verticillate (30). Fortunately, this adverse event seemed not to affect visual acuity, which could be recognized only under biomicroscopy (31). Another commonly observed ocular AE was conjunctival hemorrhage. Compared with latanoprost, FDC also resulted in a significantly higher incidence of conjunctival hemorrhage. As previously demonstrated, the inhibition of Rho kinase may result in the relaxation of vascular smooth muscle (27). Moreover, impairment of barrier function or morphologic changes were observed during the administration of Y-39983, another Rho kinase inhibitor, in human umbilical venous endothelial cells, which has been presumably due to the Rho-ROCK signaling pathway (32). We hypothesized that these effects contributed to conjunctival hemorrhage. Although other AEs, such as eye pruritus, blepharitis, increased lacrimation and blurred vision, showed a higher incidence in the FDC group than in the latanoprost group, they could not be conclusively determined to be associated with the active ingredients of FDC. Additionally, despite corneal disorders, those changes seemed to be asymptomatic, while no significant changes were observed in corneal thickness or corneal endothelial cell density, and no corneal edema was reported either (20). All the AEs reported in the existing studies were mild, and no serious treatment-related AEs were observed. Nevertheless, perhaps due to the lack of long-term observations, there are no significant systemic safety issues reported.

Several limitations should be acknowledged: (1) The number of study participants included in this study was insufficient, and limited RCTs were included. In addition, all three of the multicenter clinical trials included in the analysis were conducted in North America, which may cause potential ethnic bias. (2) Owing to the lack of long-term observations, no significant systemic safety issues were reported. Therefore, we do not know whether this novel FDC, compared with monotherapy, increases the risk of systemic side effects similar to other FDCs. (3) As the duration of follow-up was not long enough (no more than 1 year), we lacked information about the comparison of long-term efficacy between FDC and monotherapy. (4) Only POAG and OHT patients were included, and further comparison of efficacy and safety for specific patients diagnosed with other glaucoma types might be necessary. (5) We cannot absolutely rule out the potential bias presented in the results of the included studies (e.g., measurement bias). (6) Considering the promotion in less-developed regions or countries, the evaluation of cost–effectiveness is also greatly warranted. Additionally, few existing studies have elucidated the issue of whether the effects of netarsudil/latanoprost FDC at different concentrations vary, which is vital for future clinical practice. Despite several Rho-kinase inhibitors having been discovered, only two are approved for treating glaucoma (Netarsudil in the USA and Ripasudil in Japan) while no available rho-kinase inhibitor as well as related clinical trial were approved in China currently. However, as some clinical practice is well-documented, it's promising that Rho-kinase inhibitors become available commercially in China in the future.

In summary, netarsudil/latanoprost FDC appears to be better than netarsudil, with a better IOP reduction efficacy (IOP across 8:00 AM, 10:00 AM, and 4:00 PM, mean diurnal IOP, IOPR%). However, netarsudil/latanoprost FDC was associated with a significantly higher incidence of AEs, especially compared with latanoprost, which was primarily manifested in eye disorders such as conjunctival hyperemia. Furthermore, for both ocular AEs and systemic side effects, all relevant safety issues were of mild severity, and most of them were self-limiting. Therefore, we believe that the safety of netarsudil/latanoprost FDC was acceptable. Overall, as demonstrated in the present study, with the help of this novel netarsudil/latanoprost FDC, the improvements in treatment adherence and efficacy in patients with glaucoma are promising.

## Data availability statement

The original contributions presented in the study are included in the article/Supplementary material, further inquiries can be directed to the corresponding author/s.

## Author contributions

WZ had full access to all of the data in the manuscript and takes responsibility for the integrity of the data and the accuracy of the data analysis. NL, XJ, MH, and ZF: drafting of the manuscript. NL and WZ: critical revision of the manuscript for important intellectual content and statistical analysis. NL, YW, and WZ: supervision. All authors: concept and design and acquisition, analysis, or interpretation of data. All authors read and approved the final manuscript.

## Funding

This study was supported by Natural Science Foundation of Jiangxi Province (Grant No: 20212BAB206050), Science and technology planning project of Health Commision of Jiangxi Province (Grant No: 202110045), and Science and technology planning project of Jiangxi Administration of traditional Chinese Medicine (Grant No: 2020B0108). The funding had no role in the design and conduct of the study; collection, management, analysis, and interpretation of the data; preparation, review, or approval of the manuscript; and decision to submit the manuscript for publication.

## Conflict of interest

The authors declare that the research was conducted in the absence of any commercial or financial relationships that could be construed as a potential conflict of interest.

## Publisher's note

All claims expressed in this article are solely those of the authors and do not necessarily represent those of their affiliated organizations, or those of the publisher, the editors and the reviewers. Any product that may be evaluated in this article, or claim that may be made by its manufacturer, is not guaranteed or endorsed by the publisher.

## Supplementary material

Supplementary Figure 1 Qualitative assessment of RCTs with the risk-of-bias tool outlined in the Cochrane Handbook.

Supplementary Figure 2 Forest plots of RR of total adverse events associated with FDC vs. monotherapy.

Supplementary Figure 3 Forest plots of RR of general disorders and administration site conditions associated with FDC vs. monotherapy.

Supplementary Figure 4 Forest plots of RR of investigations associated with FDC vs. monotherapy.

Supplementary Figure 5 Forest plots of RR of infections and infestations associated with FDC vs. monotherapy.

Supplementary Figure 6 Forest plots of RR of eye disorders associated with FDC vs. monotherapy.

Supplementary Figure 7 Meta-based influence analysis of mean diurnal IOP **(A)** and IOPR% **(B)** for the comparison between Netarsudil and FDC and mean diurnal IOP **(C)** and IOPR% **(D)** for the comparison between Latanoprost and FDC.

Supplementary Figure 8 Begg and Egger tests of mean diurnal IOP **(A)** and IOPR% **(B)** for the comparison between Netarsudil and FDC and mean diurnal IOP **(C)** and IOPR% **(D)** for the comparison between Latanoprost and FDC.

Supplementary Table 1 Search strategy.

Supplementary Table 2 Quality assessment of all included studies.

Supplementary Table 3 GRADE Quality assessment by therapeutic strategy and study design for the outcomes of IOP across diurnal time points, mean diurnal IOP, IOPR% and adverse events.

## Abbreviations

AEs: Adverse events
CI: Confidence interval
FDA: Food and Drug Administration
FDC: Fixed-dose combination
GRADE, Grading of recommendations assessment, development: and evaluation
IOP: Intraocular pressure
IOPR%: Percentage intraocular pressure reduction
MD: Mean difference
OAG: Open-angle glaucoma
OHT: Ocular hypertension
PG: Prostaglandin
PRISMA: Preferred reporting items for systematic review and Meta-Analysis
RCT: Randomized controlled trial
RR: Risk ratios
VS: versus.
