# Supplementary figures and images for: Efficacy and safety of netarsudil/latanoprost fixed-dose combination vs. monotherapy in open-angle glaucoma or ocular hypertension: A systematic review and meta-analysis of randomized controlled trials
Source: Front Med (Lausanne). 2022 Aug 1;9:923308. doi: 10.3389/fmed.2022.923308 (PMC9376331; doi:10.3389/fmed.2022.923308)

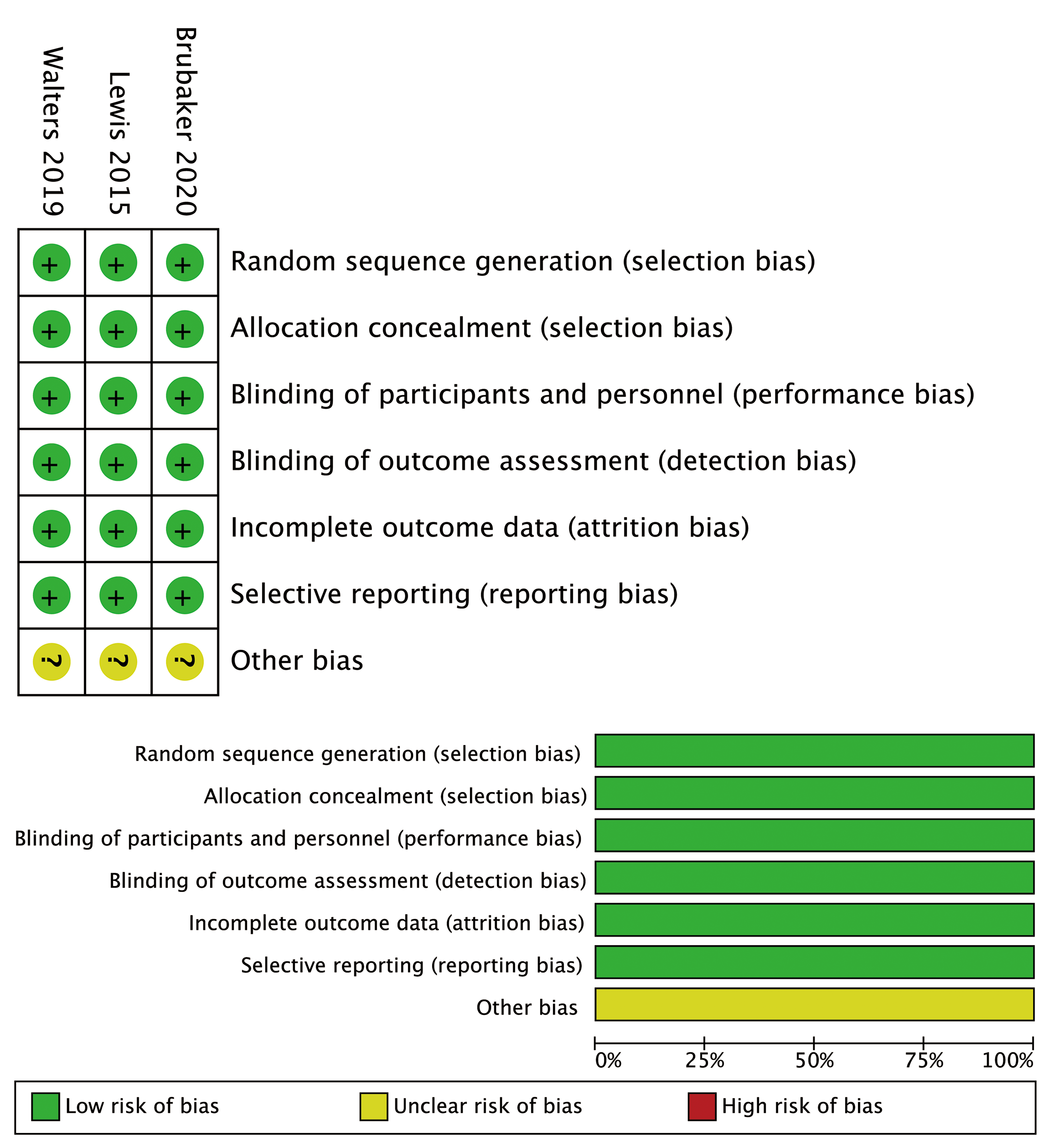

Supplement: Supplementary Figure 1 — Qualitative assessment of RCTs with the risk-of-bias tool outlined in the Cochrane Handbook. [file Image_1.TIF]

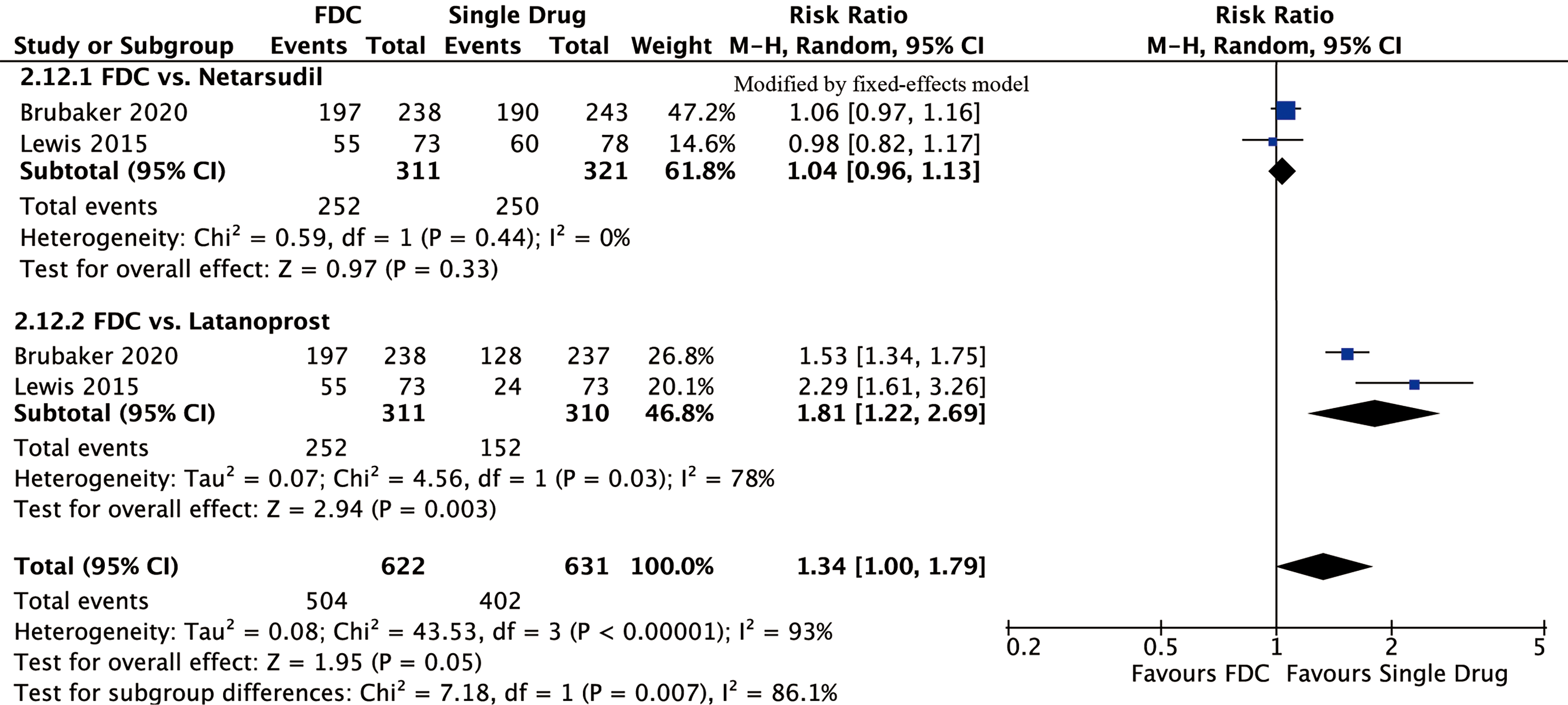

Supplement: Supplementary Figure 2 — Forest plots of RR of total adverse events associated with FDC vs. monotherapy. [file Image_2.TIF]

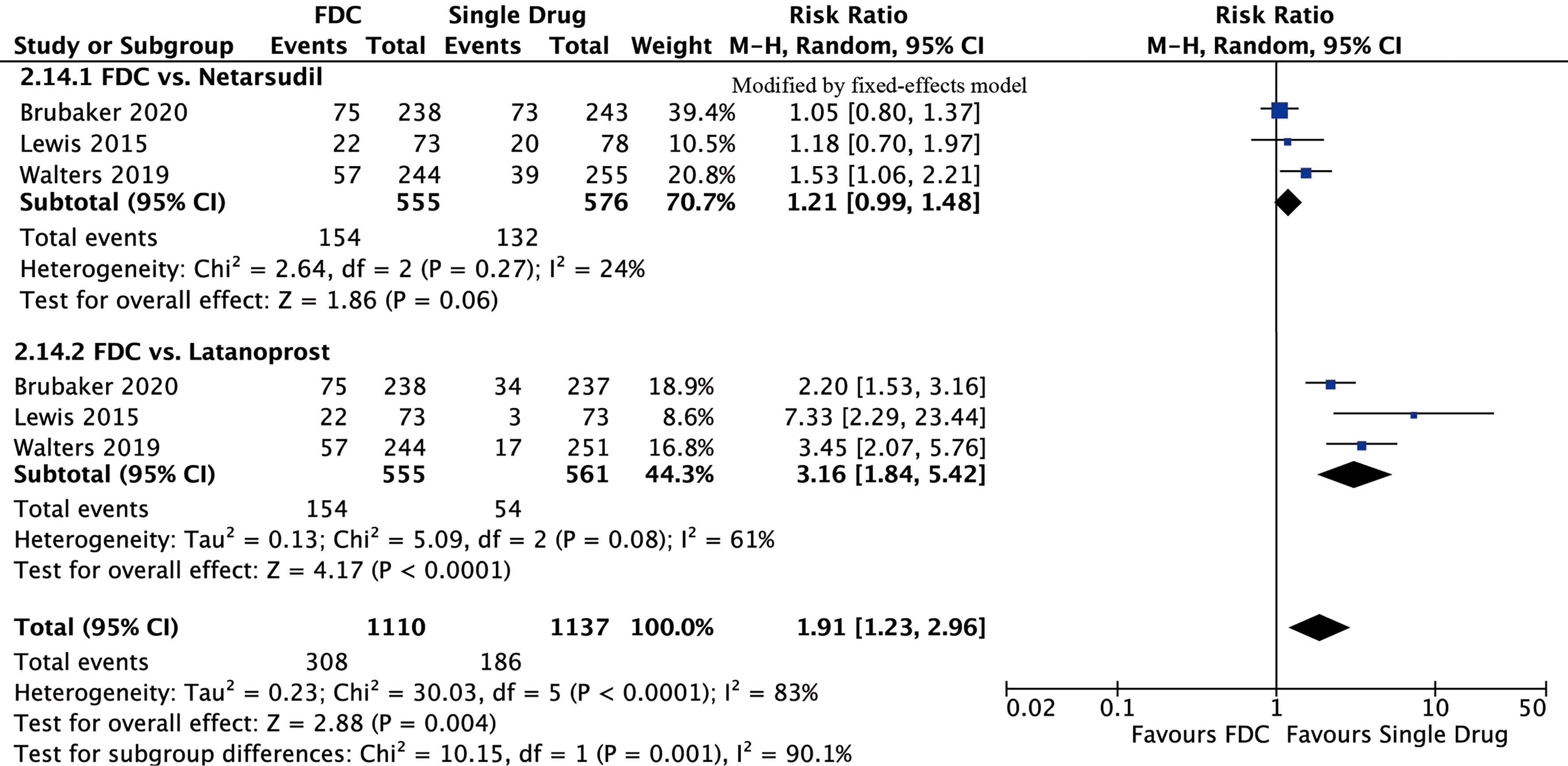

Supplement: Supplementary Figure 3 — Forest plots of RR of general disorders and administration site conditions associated with FDC vs. monotherapy. [file Image_3.TIF]

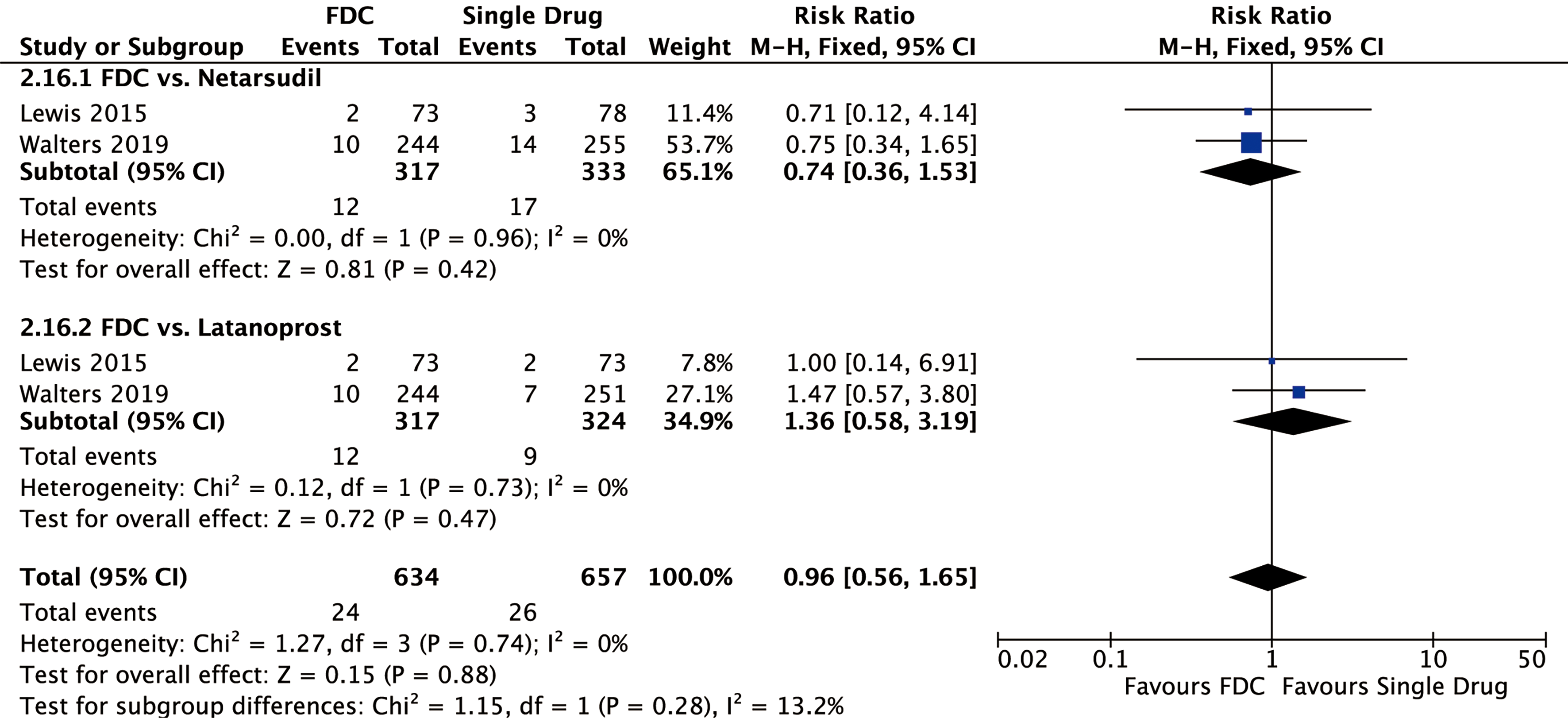

Supplement: Supplementary Figure 4 — Forest plots of RR of investigations associated with FDC vs. monotherapy. [file Image_4.TIF]

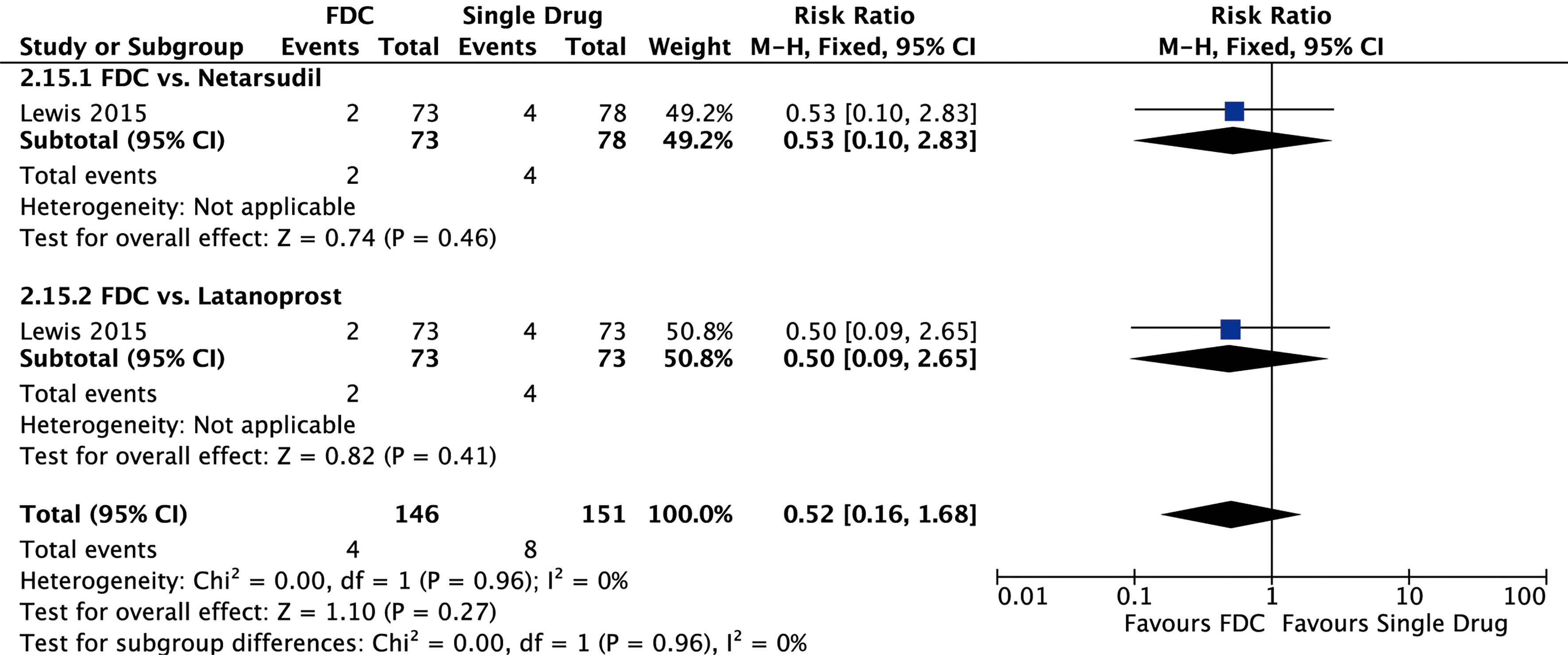

Supplement: Supplementary Figure 5 — Forest plots of RR of infections and infestations associated with FDC vs. monotherapy. [file Image_5.TIF]

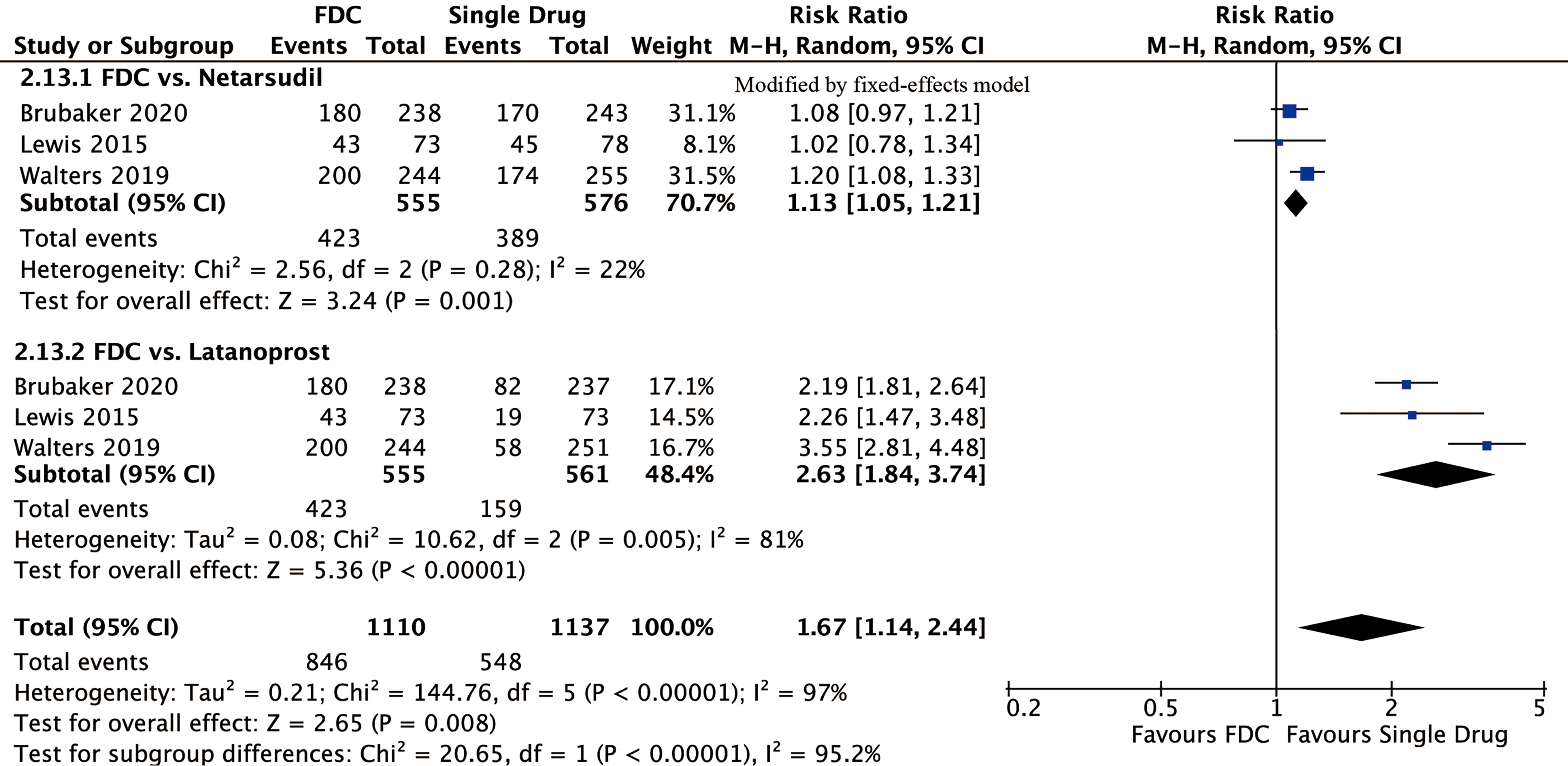

Supplement: Supplementary Figure 6 — Forest plots of RR of eye disorders associated with FDC vs. monotherapy. [file Image_6.TIF]

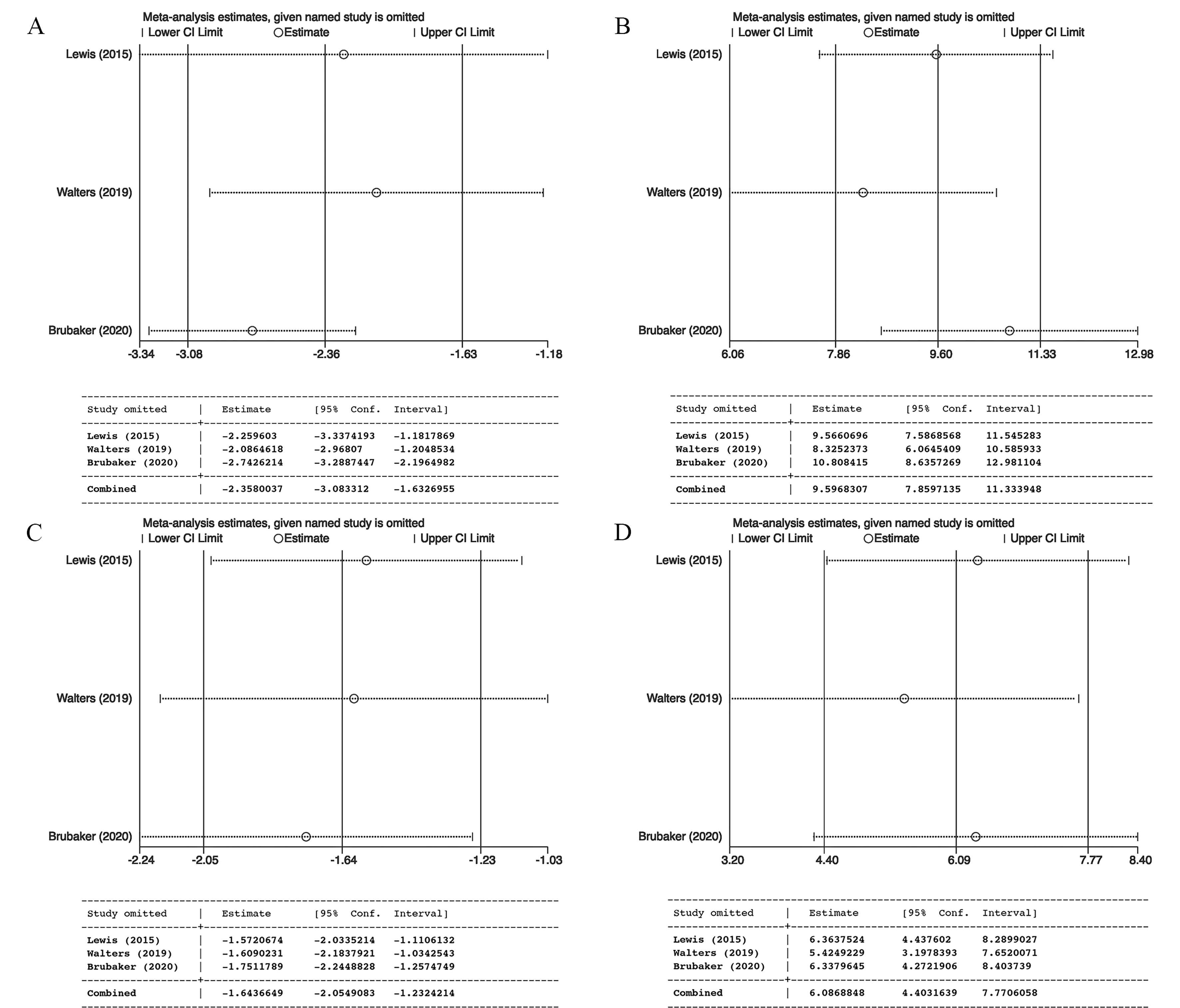

Supplement: Supplementary Figure 7 — Meta-based influence analysis of mean diurnal IOP (A) and IOPR% (B) for the comparison between Netarsudil and FDC and mean diurnal IOP (C) and IOPR% (D) for the comparison between Latanoprost and FDC. [file Image_7.TIF]

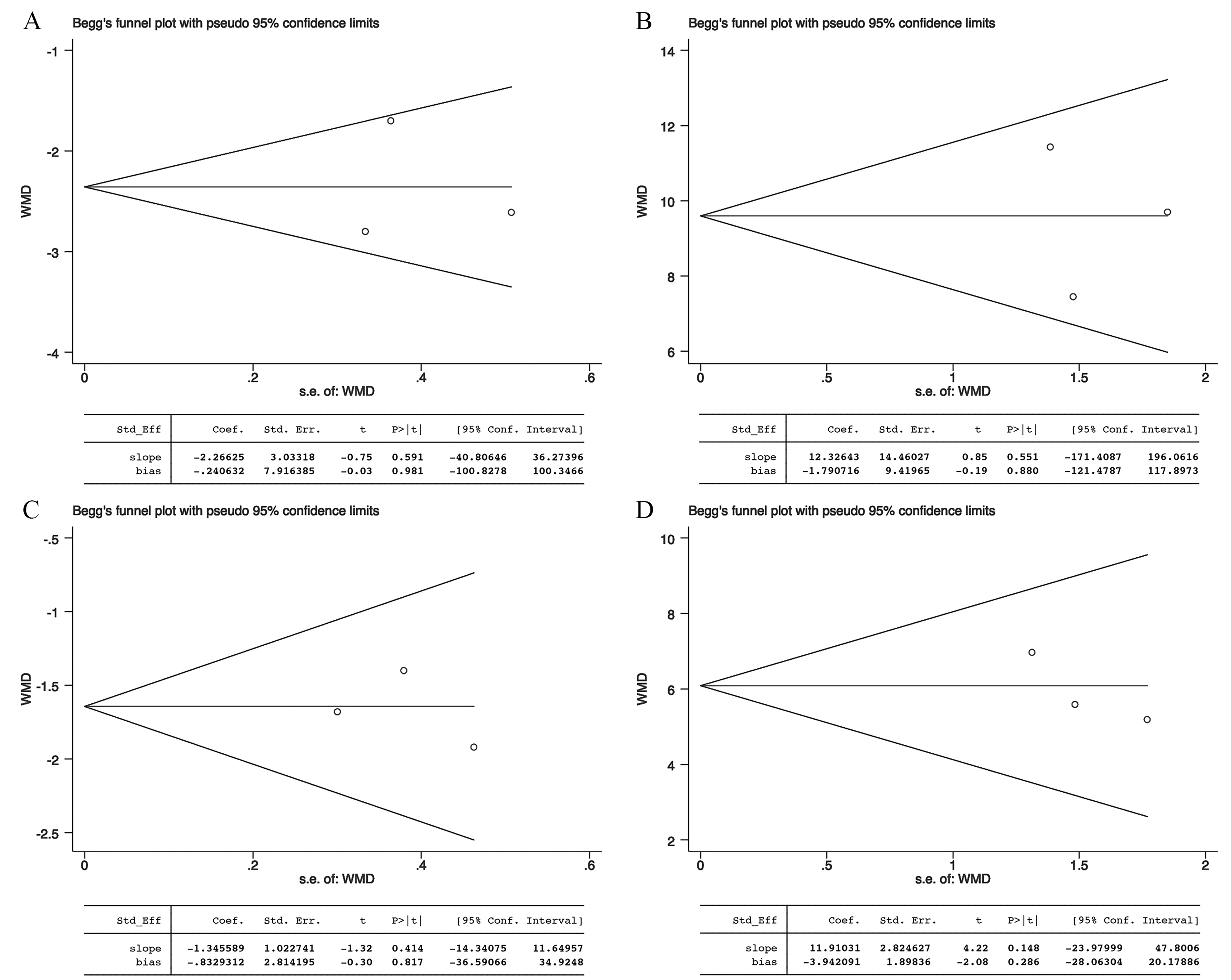

Supplement: Supplementary Figure 8 — Begg and Egger tests of mean diurnal IOP (A) and IOPR% (B) for the comparison between Netarsudil and FDC and mean diurnal IOP (C) and IOPR% (D) for the comparison between Latanoprost and FDC. [file Image_8.TIF]
