# Supplementary material for: Efficacy and safety of netarsudil/latanoprost fixed-dose combination vs. monotherapy in open-angle glaucoma or ocular hypertension: A systematic review and meta-analysis of randomized controlled trials
Source: Front Med (Lausanne). 2022 Aug 1;9:923308. doi: 10.3389/fmed.2022.923308 (PMC9376331; doi:10.3389/fmed.2022.923308)
Supplement: Supplementary Table 1 — Search strategy. [file Table_1.docx]

**Table S1** Search strategy

| **PubMed**  The database was searched on Jan 25, 2021, n=26.  Search Strategy:  ("Glaucoma"[Title/Abstract] OR "Glaucomas"[Title/Abstract] OR "Glaucoma"[MeSH Terms]) AND ("PhXA34"[Title/Abstract] OR "PHXA41"[Title/Abstract] OR "Xalatan"[Title/Abstract] OR "Latanoprost"[Title/Abstract] OR "Latanoprost"[MeSH Terms]) AND ("Netarsudil"[Title/Abstract] OR "AR-13324"[Title/Abstract] OR "Netarsudil"[Supplementary Concept]) |
| --- |
| **Web of Science**  The database was searched on Jan 25, 2021, n=38.  Search Strategy:  1 TOPIC: (“Glaucoma”) (66964)  2 TOPIC: (“Latanoprost” OR “PhXA34” OR “PHXA41” OR “Xalatan”) (20703)  3 TOPIC: (“Netarsudil” OR “AR-13324”) (108)  4 #1 AND #2 AND #3 (38) |
| **EMBASE**  The database was searched on Jan 25, 2021, n=29.  Search Strategy:  ('Glaucoma':ti,ab,kw) AND ('Latanoprost':ti,ab,kw OR 'PhXA34':ti,ab,kw OR 'PHXA41':ti,ab,kw OR 'Xalatan':ti,ab,kw) AND ('Netarsudil':ti,ab,kw OR 'AR-13324':ti,ab,kw) |
| **Cochrane Library**  The database was searched on Jan 25, 2021, n=23.  Search Strategy:  (“Glaucoma”): ti,ab,kw AND (“Latanoprost” OR “PhXA34” OR “PHXA41” OR “Xalatan”): ti,ab,kw AND (“Netarsudil” OR “AR-13324”): ti,ab,kw - (Word variations have been searched) |
| **Ovid MEDLINE**  The database was searched on Jan 25, 2021, n=17.  Search Strategy:  1 Glaucoma.ab. (41897)  2 Latanoprost.ab. (1506)  3 PhXA34.ab. (10)  4 PHXA41.ab. (27)  5 Xalatan.ab. (133)  6 Netarsudil.ab. (51)  7 AR-13324.ab. (11)  8 or/2-5 [Latanoprost] (1554)  9 or/6-7 [Netarsudil] (58)  10 1 and 8 and 9 (17) |
| **ScienceDirect**  The database was searched on Jan 25, 2021, n=2.  Search Strategy:  Title, abstract, keywords: ((“Glaucoma”) and (“Latanoprost” OR “PhXA34” OR “PHXA41” OR “Xalatan”) and (“Netarsudil” OR “AR-13324”)) |

**Note:** The combined text and medical subject heading (MeSH) terms used were: “Glaucoma”, “Latanoprost” and “Netarsudil”.
