# Supplementary material for: Efficacy and safety of netarsudil/latanoprost fixed-dose combination vs. monotherapy in open-angle glaucoma or ocular hypertension: A systematic review and meta-analysis of randomized controlled trials
Source: Front Med (Lausanne). 2022 Aug 1;9:923308. doi: 10.3389/fmed.2022.923308 (PMC9376331; doi:10.3389/fmed.2022.923308)
Supplement: Supplementary Table 2 — Quality assessment of all included studies. [file Table_2.docx]

**Table S2** Quality assessment of all included studies

| **Study** | | **Randomization** | **Masking** | **Accountability of all patients** | **Quality (score)** |
| --- | --- | --- | --- | --- | --- |
| 2020 | Brubaker [18] | ★★ | ★★ | ★ | 5 |
| 2015 | Lewis [19] | ★★ | ★★ | ★ | 5 |
| 2019 | Walters [20] | ★★ | ★★ | ★ | 5 |
