# Supplementary material for: Efficacy and safety of netarsudil/latanoprost fixed-dose combination vs. monotherapy in open-angle glaucoma or ocular hypertension: A systematic review and meta-analysis of randomized controlled trials
Source: Front Med (Lausanne). 2022 Aug 1;9:923308. doi: 10.3389/fmed.2022.923308 (PMC9376331; doi:10.3389/fmed.2022.923308)
Supplement: Supplementary Table 3 — GRADE Quality assessment by therapeutic strategy and study design for the outcomes of IOP across diurnal time points, mean diurnal IOP, IOPR% and adverse events. [file Table_3.docx]

**Table S3** GRADE Quality assessment by therapeutic strategy and study design for the outcomes of IOPR efficacy and adverse events.

| **Primary outcomes** | | **Comparison** | **No. of Studies** | **No. of participants** | | **Differences^a^（95%CI）** | **Quality assessment** | | | | | **Quality** |
| --- | --- | --- | --- | --- | --- | --- | --- | --- | --- | --- | --- | --- |
|  |  |  |  | **FDC** | **Single Drug** |  | **Risk of bias^b^** | **Inconsistency** | **Indirectness** | **Imprecision** | **Publication bias^c^** |  |
| **IOPR Efficacy** |  | |  |  |  |  |  |  |  |  |  |  |
| Mean Diurnal IOP | FDC vs. Netarsudil | | 3 | 555 | 577 | -2.36 [-3.08, -1.63] | Low | No inconsistency | No indirectness | No imprecision | Unlikely | High |
|  | FDC vs. Latanoprost | | 3 | 555 | 559 | -1.64 [-2.05, -1.23] | Low | No inconsistency | No indirectness | No imprecision | Unlikely | High |
| IOP |  | |  |  |  |  |  |  |  |  |  |  |
| 8:00 | FDC vs. Netarsudil | | 3 | 555 | 577 | -2.73 [-3.96, -1.49] | Low | No inconsistency | No indirectness | No imprecision | Unlikely | High |
|  | FDC vs. Latanoprost | | 3 | 555 | 559 | -1.54 [-1.99, -1.09] | Low | No inconsistency | No indirectness | No imprecision | Unlikely | High |
| 10:00 | FDC vs. Netarsudil | | 3 | 555 | 577 | -2.43 [-2.84, -2.01] | Low | No inconsistency | No indirectness | No imprecision | Unlikely | High |
|  | FDC vs. Latanoprost | | 3 | 555 | 559 | -1.74 [-2.14, -1.34] | Low | No inconsistency | No indirectness | No imprecision | Unlikely | High |
| 16:00 | FDC vs. Netarsudil | | 3 | 555 | 577 | -1.93 [-2.32, -1.53] | Low | No inconsistency | No indirectness | No imprecision | Unlikely | High |
|  | FDC vs. Latanoprost | | 3 | 555 | 559 | -1.57 [-1.95, -1.18] | Low | No inconsistency | No indirectness | No imprecision | Unlikely | High |
| IOPR% | FDC vs. Netarsudil | | 3 | 555 | 577 | 9.60 [7.86, 11.33] | Low | No inconsistency | No indirectness | No imprecision | Unlikely | High |
|  | FDC vs. Latanoprost | | 3 | 555 | 559 | 6.09 [4.40, 7.77] | Low | No inconsistency | No indirectness | No imprecision | Unlikely | High |
| **Adverse events** |  | |  |  |  |  |  |  |  |  |  |  |
| Any Adverse Event | FDC vs. Netarsudil | | 2 | 252/311 | 250/321 | 1.04 [0.96, 1.13] | Low | No inconsistency | No indirectness | Serious (-1) | Unlikely | Moderate |
|  | FDC vs. Latanoprost | | 2 | 252/311 | 152/310 | 1.81 [1.22, 2.69] | Low | No inconsistency | No indirectness | Serious (-1) | Unlikely | Moderate |
| Eye Disorders | FDC vs. Netarsudil | | 3 | 423/555 | 389/576 | 1.13 [1.05, 1.21] | Low | No inconsistency | No indirectness | No imprecision | Unlikely | High |
|  | FDC vs. Latanoprost | | 3 | 423/555 | 159/561 | 2.63 [1.84, 3.74] | Low | No inconsistency | No indirectness | No imprecision | Unlikely | High |
| General Disorders and | FDC vs. Netarsudil | | 3 | 154/555 | 132/576 | 1.21 [0.99, 1.48] | Low | No inconsistency | No indirectness | Serious (-1) | Unlikely | Moderate |
| Administration Site Conditions | FDC vs. Latanoprost | | 3 | 154/555 | 54/561 | 3.16 [1.84, 5.42] | Low | No inconsistency | No indirectness | No imprecision | Unlikely | High |
| Infections and infestations | FDC vs. Netarsudil | | 1 | 2/73 | 4/78 | 0.53 [0.10, 2.83] | Low | No inconsistency | No indirectness | Very Serious (-2) | Unlikely | Low |
|  | FDC vs. Latanoprost | | 1 | 2/73 | 4/73 | 0.50 [0.09, 2.65] | Low | No inconsistency | No indirectness | Very Serious (-2) | Unlikely | Low |
| Investigations | FDC vs. Netarsudil | | 2 | 12/317 | 17/333 | 0.74 [0.36, 1.53] | Low | No inconsistency | No indirectness | Serious (-1) | Unlikely | Moderate |
|  | FDC vs. Latanoprost | | 2 | 12/317 | 9/324 | 1.36 [0.58, 3.19] | Low | No inconsistency | No indirectness | Serious (-1) | Unlikely | Moderate |

**Abbreviations:** CI: confidence interval; FDC: fixed-dose combination; IOP: intraocular pressure; IOPR: intraocular pressure reduction; IOPR%: intraocular pressure reduction percentage; vs.: versus.

^a^ Differences: risk ratios (RR) for adverse events; weighted mean difference (WMD) for IOPR efficacy.

^b^ Risk of bias assessed using the Cochrane risk of bias (ROB) tool for RCTs.

^c^ Publication bias was assessed by Egger’s and Begg’s tests.
